# Combination Therapy with Cholinesterase Inhibitors and Memantine for Alzheimer’s Disease: A Systematic Review and Meta-Analysis

**DOI:** 10.1093/ijnp/pyu115

**Published:** 2015-03-05

**Authors:** Shinji Matsunaga, Taro Kishi, Nakao Iwata

**Affiliations:** Department of Psychiatry, Fujita Health University School of Medicine, Toyoake, Aichi, Japan.

**Keywords:** Alzheimer’s disease, memantine, cholinesterase inhibitors, systematic review, meta-analysis

## Abstract

**Background:**

We performed an updated meta-analysis of randomized controlled trials of combination therapy with cholinesterase inhibitors and memantine in patients with Alzheimer’s disease.

**Methods:**

We reviewed cognitive function, activities of daily living, behavioral disturbance, global assessment, discontinuation rate, and individual side effects.

**Results:**

Seven studies (total n=2182) were identified. Combination therapy significantly affected behavioral disturbance scores (standardized mean difference=−0.13), activity of daily living scores (standardized mean difference=−0.10), and global assessment scores (standardized mean difference=−0.15). In addition, cognitive function scores (standardized mean difference=−0.13, *P*=.06) exhibited favorable trends with combination therapy. The effects of combination therapy were more significant in the moderate-to-severe Alzheimer’s disease subgroup in terms of all efficacy outcome scores. The discontinuation rate was similar in both groups, and there were no significant differences in individual side effects.

**Conclusions:**

Combination therapy was beneficial for the treatment of moderate-to-severe Alzheimer’s disease in terms of cognition, behavioral disturbances, activities of daily living, and global assessment was well tolerated.

## Introduction

Dementia is not only a health problem but also a social problem. Alzheimer’s Disease International reported that over 35 million people currently live with dementia (International AsD, 2009). In Japan, the prevalence rate of dementia among those aged ≥65 years is estimated to be 15.8%, with Alzheimer’s disease (AD) being the most common cause and accounting for 65.8% of its global incidence (Asada, 2012). AD is a neurodegenerative disease characterized by progressive loss of cognition and other neurobehavioral symptoms. The pathology of AD includes extracellular senile plaques primarily consisting of β-amyloid and intracellular neurofibrillary tangles consisting of abnormally hyperphosphorylated tau, which is a microtubule-associated protein (Ittner and Gotz, 2011).

Currently, cholinesterase inhibitors (ChEIs) and memantine are available for the treatment of AD. The Food and Drug Administration approves the ChEIs donepezil, galantamine, and rivastigmine for the treatment of AD. The Food and Drug Administration also approves memantine for the treatment of moderate-to-severe AD. Memantine is postulated to exert its therapeutic effect through its action as a low-to-moderate affinity, noncompetitive (open-channel) *N*-methyl-d-aspartate (NMDA) receptor antagonist, which binds preferentially to NMDA receptor-operated calcium channels (Berman et al., 2012; Kishi and Iwata, 2013). Memantine blocks the effects of sustained, pathologically elevated levels of glutamate that may lead to neuronal dysfunction (Danysz and Parsons, 2003).

A recent meta-analysis (3 studies, 971 patients; Muayqil and Camicioli, 2012) suggested that combination therapy with ChEI and memantine (ChEI+MEM) showed a significant effect size for moderate-to-severe AD in terms of cognitive function (standardized mean difference [SMD]=−0.45, *P=*.00001) and the neuropsychiatric inventory [NPI; 7991117; mean difference=4.40, *P=.*00001]. However, the number of studies and patients included in the meta-analysis were small. The limitation of a meta-analysis with small samples is the possibility of statistical errors because of low statistical power. Therefore, we have updated the meta-analysis of ChEI+MEM for AD (current meta-analysis: 7 studies, 2182 patients). To our knowledge, 7 randomized controlled trials (RCTs) concerning ChEI+MEM for AD have been performed to date (Tariot et al., 2004; Cretu et al., 2008; Porsteinsson et al., 2008; Choi et al., 2011; Howard et al., 2012; Grossberg et al., 2013; Dysken et al., 2014).

Cognitive function has been considered in 2 studies (Tariot et al., 2004; Grossberg et al., 2013) reporting that ChEI+MEM was superior to placebo using the Severe Impairment Battery (SIB; Panisset et al., 1994). Other studies have shown that ChEI+MEM was not superior to placebo (Porsteinsson et al., 2008; Dysken et al., 2014) or usual ChEI therapy (Choi et al., 2011) using the Alzheimer’s Disease Assessment Scale cognitive subscale (ADAS-cog; Rosen et al., 1984), whereas in one study, the ADAS-cog statistical result was unknown (Cretu et al., 2008). Another study (Howard et al., 2012) showed that ChEI+MEM was not superior to placebo in the standardized Mini-Mental State Examination (SMMSE; Molloy and Standish, 1997). With regard to behavioral disturbance, 3 studies (Tariot et al., 2004; Howard et al., 2012; Grossberg et al., 2013) reported that ChEI+MEM was superior to placebo using the NPI (Cummings et al., 1994). Other studies have reported that ChEI+MEM was not superior to either placebo (Porsteinsson et al., 2008; Dysken et al., 2014) or usual ChEI therapy (Choi et al., 2011) using the NPI, while the NPI statistical result was unknown in another study (Cretu et al., 2008). As shown by the above results, these discrepant results may be due to the small sample sizes in the trials. A meta-analysis produces a weighted summary result (more weight given to larger studies). By combining results from more than one study, a meta-analysis has the advantage of increasing statistical power, which is often inadequate in studies with a small sample size (Cohn and Becker, 2003). Moreover, we can combine outcomes with different measurements using SMD analyses (DerSimonian and Laird, 1986). To clarify whether ChEI+MEM is more efficacious in terms of several outcomes and safer than ChEI monotherapy in patients with AD, we performed an updated meta-analysis of ChEI+MEM in patients with AD.

## Methods

A meta-analysis was conducted according to the guidelines from the Preferred Reporting Items for Systematic Reviews and Meta-Analyses group (Moher et al., 2009).

### Inclusion Criteria, Search Strategy, Data Extraction, and Outcome Measures

We included RCTs of ChEI+MEM for patients with AD in this study. We selected only those RCTs that used combination therapy with ChEI in patients with AD and allowed the inclusion of studies that were not double-blinded and not placebo-controlled (ie, treatment as usual) in order to include more studies. To identify relevant studies, we searched PubMed, the Cochrane Library databases, Google Scholar, EMBASE, CINAHL, and PsycINFO citations. There were no language restrictions, and we considered all studies published up to October 22, 2014. We used the following keywords: “donepezil,” “rivastigmine,” “galantamine,” or “cholinesterase inhibitors” AND “memantine” AND “randomized,” “random,” OR “randomly,” AND “Alzheimer’s disease,” OR “Alzheimer disease.” Additional eligible studies were sought via a search of the reference lists from primary articles and relevant reviews.

The first 2 authors of this review (S.M. and T.K.) scrutinized the inclusion and exclusion criteria for the identified studies. When data required for the meta-analysis were missing, the first and/or corresponding authors were contacted for additional information, including endpoint scores. The 3 authors of this study independently extracted, checked, and entered the data into Review Manager (Version 5.2 for Windows, Cochrane Collaboration, http://ims.cochrane.org/revman).

### Data Synthesis and Statistical Analysis

We included the outcome measures of at least 3 studies per outcome. The primary outcome measures for efficacy were cognitive function and behavioral disturbances associated with AD. Cognitive function was measured using the SIB, ADAS-cog, SMMSE, and MMSE (Folstein et al., 1975). Moreover, 3 studies used 2 cognitive functional scales (ADAS-cog and MMSE); in these instances, we performed pattern analyses for both scales. Behavioral disturbances were measured using the NPI. Secondary outcome measures included activities of daily living [the Alzheimer’s Disease Cooperative Study-Activities of Daily Living 23 Items (Galasko et al., 1997) and the Bristol Activities of Daily Living Scale (Bucks et al., 1996)], global assessment [the Clinician’s Interview-Based Impression of Change Plus caregiver input (Olin et al., 1996) and the Clinical Dementia Rating scale (Morris, 1993)], discontinuation for any cause, discontinuation because of adverse events, and discontinuation because of inefficacy. In addition, we pooled the side effects data.

We based our analyses on intent-to-treat or modified intent-to-treat data (ie, at least 1 dose or at least 1 follow-up assessment). However, completer analysis data were not excluded to ensure that as much information as possible was available [(Howard et al., 2012): SMMSE, NPI, and Bristol Activities of Daily Living Scale scores]. The meta-analysis was performed using Review Manager. For continuous data, the SMD was used, combining the effect-size data (Hedges’ *g*). For dichotomous data, the relative risk was estimated along with 95% confidence intervals (CIs). We explored study heterogeneity using the *I* ^*2*^ statistic, with values of ≥50% reflective of considerable heterogeneity (Higgins et al., 2003). Overall SMDs or relative risks and their 95% CIs were estimated using DerSimonian–Laird random-effects models (Higgins et al., 2003). The random-effects model is more conservative than the fixed-effects model and produces a wider CI.

In cases with *I* ^*2*^ values ≥50% for primary outcome measures, we planned to conduct sensitivity analyses to determine the reasons for heterogeneity. However, because no significant heterogeneity was found within the primary outcomes, these analyses were not conducted. Funnel plots were inspected visually to assess the possibility of publication bias. We also assessed the methodological qualities of the articles included in the meta-analysis on the basis of the Cochrane risk of bias criteria (Cochrane Collaboration; http://www.cochrane.org/).

## Results

### Study Characteristics

The search yielded a total of 431 references (duplication=313 references). Seven RCTs concerning ChEI+MEM were included in the current meta-analysis; we excluded 80 references after reviewing the title and abstract. A further 31 references were excluded after full-text reviews, because 14 were review papers, 7 were included in the current meta-analysis, 7 did not involve combination therapy, 2 were non-RCTs, and another did not concern AD. In total, we identified 2182 patients with AD across 7 RCTs that met our inclusion criteria (Tariot et al., 2004; Cretu et al., 2008; Porsteinsson et al., 2008; Choi et al., 2011; Howard et al., 2012; Grossberg et al., 2013; Dysken et al., 2014). Of these 7 RCTs, 3 concerned ChEI+MEM, 3 concerned donepezil and memantine, and 1 concerned a rivastigmine patch and memantine.

The mean study duration was 27 weeks, with 4 trials lasting 24 weeks and 1 each lasting 52 weeks and 16 weeks. One trial was duration of study ranged from 6 months to 4 years. The total sample sizes ranged from 43 to 677 patients in each study. The mean age of the study population was 76 years. Four of 7 studies were sponsored by the pharmaceutical industry and 1 of 7 studies was published in Romanian (Cretu et al., 2008). The studies were conducted in 1 or multiple countries: 3 were conducted in the United States, 1 was conducted in South Korea, 1 was conducted in the United Kingdom, 1 was conducted in Romania, and 1 was conducted in Argentina, Chile, Mexico, and the United States. The characteristics of the trials included in our study are shown in Table 1.

**Table 1.** Characteristics of Included Trials

Study	Total n	Patients	Diagnosis	Duration	Age (mean ± SD)	Male %	Race (%)	Drug	n	Dose (dose mg/day)	With ChEI % (mean or median dose, ± SD mg)	Outcomes
Tariot 2004 (USA) industry	404	AD: Outpatient (NR) Inclusions: age ≥50 y, MMSE 5–14, MRI or CT consistent with a diagnosis of probable AD (within 12 mo), ongoing DON therapy for >6 mo before entrance into the trial and at a stable dose (5–10mg/d) for at least 3 months, caregiver to accompany the patient to research visits and oversee the administration of the investigational agent during the trial, residence in the community, ambulatory or ambulatory-aided ability, stable medical condition, permitted to continue receiving stable doses of concomitant medication (including antidepressants, antihypertensive, antiinflammatory drugs, atypical antipsychotics, antiparkinsonian drugs, laxatives, diuretics, and sedatives/hypnotics.), Exclusions: B12 or folate deficiency, active disease (pulmonary, gastrointestinal, renal, hepatic, endocrine, or cardiovascular disease.), other psychiatric or central nervous system disorders, dementia complicated by other organic disease, modified HIS 4 <.	probable AD: NINCDS-ADRDA criteria	24 wk	MEM (75.5 ± 8.45); PLA (75.5 ± 8.73)	MEM, 37; PLA, 33	MEM: White (90.1); PLA: White (92.5)	MEM	203	MEM 20mg, [fixed]	DON 100 % (9.25±1.79)	MEM>PLA: SIB, ADCS-ADL, CIBIC-Plus, NPI, BGP
								PLA	201	PLA	DON 100 % (9.49±1.88)	
Porsteinsson 2008 (USA) industry	433	AD: Outpatient (NR) Inclusions: age ≥50 years, MMSE 10–22, MRI or CT consistent with a diagnosis (within 12 mo), treatment with a ChEI for 6 mo or longer, and a stable dosing regimen for 3 mo or longer (DON 5 or 10mg/d, RIV 6,9, or 12mg/d, GAL 16 or 24mg/d), caregiver to accompany the participant to all study visits and supervise administration of the study drug, ability to ambulate, vision and hearing sufficient to permit compliance with assessments, MADRS <22, medical stability. Exclusions: B12 or folate deficiency, clinically significant and active disease (pulmonary, gastrointestinal, renal, hepatic, endocrine, or cardiovascular disease.), other psychiatric or central nervous system disorders, dementia complicated by other organic disease or AD with delusions or delirium, undergoing treatment for an oncology diagnosis, or completion of treatment within 6 mo of screening, modified HIS 4 <, poorly controlled hypertension, substance abuse, participation in an investigational drug study or use of an investigational drug within 30 d (or 5 half-lives, whichever is longer) of screening, depot neuroleptic use within 6 mo of screening, positive urine drug test, likely institutionalization during the trial, previous MEM treatment or participation in an investigational study of MEM, and likely cessation of ChEI treatment during the trial.	probable AD: NINCDS-ADRDA criteria	24 wk	MEM (74.9 ± 7.64); PLA (76.0 ± 8.43)	MEM, 46.1; PLA, 49.5	NR	MEM	217	MEM 20mg, (dose adjustments were permitted)	DON 71 % (9.5 ± 1.5); RIV 15.2 % (9.2 ± 2.8); GAL 13.8 % (19.7 ± 4.6)	MEM=PLA: ADAS-cog, CIBIC-Plus, ADCS-ADL, NPI, MMSE
								PLA	216	PLA	DON 63.4 % (8.9 ± 2 .1); RIV 20.4 % (10.0 ± 2.6); GAL 16.2 % (19.4 ± 5.2)	
Choi 2011 (South Korea) industry	172	AD: Outpatient (NR) Inclusions: age 50–90 y, ambulatory or ambulatory-aided, MMSE 10–20, had MRI or CT showing no clinical evidence of other diseases capable of producing a dementia syndrome, and had a reliable caregiver who met the patient at least once a week and was sufficiently familiar with the patient to provide the investigator with accurate information. Exclusions: any primary neurodegenerative disorder or psychiatric disorder other than AD, clinically significant laboratory abnormalities (such as thyroid function, B12, folate, venereal disease), any history of drug or alcohol addiction for the past 10 y, any severe or unstable medical disease, bradycardia with <50 beats/min, sick sinus syndrome, sinoatrial block, second or third degree atrioventricular block, any hearing or visual impairment that could disturb the efficient evaluation of the patient, any active skin lesion, a history of allergy to topical products containing any of the constitution of the patches, known hypersensitivity to ChEI, and an involvement in other clinical trials or treated by any experimental drug within 4wk.	probable AD: NINCDS-ADRDA criteria	16 wk	MEM (75.0 ± 7.3); UC (74.7 ± 7.7)	MEM, 25; UC, 15.7	NR	MEM	88	MEM 20mg, (dose adjustments were permitted)	RIV patch 100 % (9.77±1.041cm2)	MEM=UC: ADAS-cog, MMSE, FAB, NPI, ADCS-ADL, CDR MEM<UC: CMAI
								UC	84	UC	RIV patch 100 % (9.64±1.280cm2)	
Howard 2012 (UK) non-industry	146	AD: Outpatient (NR) Inclusions: community residents who had caregivers who either lived with them or visited them at least daily, continuously treatment with DON for at least 3 mo (treatment with DON 10mg for at least the previous 6wk), SMMSE 5–13, each eligible patient’s prescribing clinician was considering a change in drug treatment on the basis of NICE guidelines at the time, discussions with the patient and caregivers, and the physician’s clinical judgment. Exclusions: severe or unstable medical conditions, current prescription of MEM, contra-indications or previous adverse or allergic reactions to trial drugs, involvement in another trial or concerns over the patient’s compliance.	probable or possible AD: NINCDS-ADRDA criteria	52 wk	MEM (77.5 ± 9.0); PLA (77.2 ± 7.5)	MEM, 33; PLA, 30	MEM: White (92) Black (5) Other (3)PLA: White (95) Black (1) Other (4)	MEM	73	DON 10mg, MEM 20 mg	DON 100 % (10)	MEM>PLA: NPI MEM=PLA: SMMSE, BADLS, DEMQOL-proxy, GHQ-12
								PLA	73	DON 10mg, PLA	DON 100 % (10)	
Creţu 2008 (Romania) NR	43	AD: Outpatient (NR) Inclusions: age ≥50 y, MMSE 10–17, MRI or CT consistent with a diagnosis of probable AD (within 12 mo), under current treatment with DON 10mg for at least 6 mo, have a caregiver in the family who could give information about their health. Exclusions: VD, severe depressive disorder, HIS 4 <, unstable or severe somatic diseases, chronic treatment with BZD, hypnotics, anticholinergics, anxiolytics.	AD: DSM-Ⅳ-TR	24 wk	72.5	MEM, 38; UC, 37	NR	MEM UC	22 21	MEM 20 mg	DON 100 % (10)	statistical results were unknown. Favorable add-on MEM: MMSE, ADAS-cog, NPI, CMAI
Grossberg 2013 (Argentina, USA, Mexico, Chile), industry	677	AD: Outpatient (100%) Inclusions: age ≥50 y, MMSE 3–14, results of a MRI or CT consistent with a diagnosis (within the past 12 mo), ongoing CHEI therapy (stable dosage for at least 3 mo), normal (or clinically nonsignificant) results on physical examination, laboratory evaluations, and ECG. Exclusions: clinically significant and active pulmonary, gastrointestinal, renal, hepatic, endocrine, or cardiovascular system disease or cancer, a neurologic disorder or dementia complicated by other organic disease or predominant delusions, DSM-IV Axis I disorder other than AD, evidence of clinically significant disease involving the central nervous system, systolic hypertension or hypotension, HIS 4 <, and current or prior exposure to any unapproved concomitant medication that could not be discontinued or switched to an allowable alternative medication before baseline.	probable AD: NINCDS-ADRDA and DSM-IV-TR criteria	24 wk	MEM (76.2 ± 8.4); PLA (76.8 ± 7.8)	MEM, 28.4; PLA, 27.5	MEM: White (95) Hispanic (68.3) PLA: White (93.1) Hispanic (69.6)	MEM	342	MEM (28mg, extended-release)	DON 69.2 %, (8.0±2.8); RIV 9.4 %, (6.8±2.6); GAL 21.1 %, (13.5±5.7)	MEM>PLA: SIB, CIBIC-Plus, NPI, VFT MEM=PLA: ADCS-ADL
								PLA	335		DON 68.1 %, (7.8±2.6); RIV 12.2 %, (6.8±2.9); GAL 20.3 %, (13.5±5.4)	
Dysken 2014 (USA) non-industry	307	AD: Outpatient (NR) Inclusions: MMSE 12–26, presence of a caregiver who can assume responsibility for medication compliance, can accompany the patient to all visits, and rate patient’s condition, written informed consent from both the patient and caregiver, administration of maintenance dosage of a ChEI for at least 4wk. Exclusions: other psychiatric disorders, presence of any uncontrolled illness, pregnant or intention to became pregnant, use of WAR, VitE (past 2wk), MEM (past 4wk), and AMA (past 2wk), creatinine clearance 5 < mL/min.	probable or possible AD: NINCDS-ADRDA criteria	6 mo to 4 y	MEM (78.8 ± 7.2); PLA (79.4 ± 7.0)	MEM, 96; PLA, 98	MEM: White (85) Black (14) Other (1)PLA: White (86) Black (13) Other (1)	MEM	155	MEM 20mg, (dose adjustments were permitted)	DON 65 %, (NR); RIV 5 %, (NR); GAL 30 %, (NR)DON 63 %, (NR); RIV 1 %, (NR); GAL 36 %, (NR)	MEM=PLA: ADCS-ADL, ADAS-cog, NPI, MMSE, CAS
								PLA	152			

AD, Alzheimer’s Disease, ADAS-cog, Alzheimer’s Disease Assessment Scale cognitive subscale, ADCS-ADL, Alzheimer’s Disease Cooperative Study–Activities of Daily Living, ADL, activities of daily living, AMA, amantadine, BADLS, Bristol Activities of Daily Living Scale, BGP, Behavioral Rating Scale for Geriatric Patients, BZD, benzodiazepine, CAS, Caregiver Activity Survey, CDR, Clinical Dementia Rating scale, CIBIC-Plus, Clinician’s Interview-Based Impression of Change Plus Caregiver Input, ChEI, cholinesterase inhibitors, CMAI, Cohen Mansfield Agitation Inventory, CT, computed tomographic scan, d, days, DON, donepezil, DSM-IV-TR, Diagnostic and Statistical Manual of Mental Disorders-4th edition-text revision, ECG, electrocardiogram, FAB, Frontal Assessment Battery, GAL, galantamine, GHQ-12, General Health Questionnaire 12, HIS, Hachinski Ischaemic Score, MADRS, Montgomery-Asberg Depression Rating Scale, MMSE, Mini-Mental State Examination, MRI, magnetic resonance imaging, m, months, NICE, National Institute for Health and Clinical Excellence, NINCDS-ADRDA, National Institute of Neurological and Communicative Disorders and Stroke and the Alzheimer’s Disease and Related Disorders Association, NPI, neuro-psychiatric inventory, NR, Not report, RIV, rivastigmine, SIB, Severe Impairment Battery, SMMSE, Standardized Mini–Mental State Examination, UC, usual care, VD, vascular dementia, VFT, verbal fluency test, VitE, vitamin E, WAR, warfarin, y, years.

We evaluated the methodological quality of all studies using the Cochrane risk of bias criteria. Five of the 7 studies were double-blind, placebo-controlled trials and mentioned the required details of study design. Another study was an open-label, randomized, non-placebo-controlled trial. The study published by Cretu and colleagues (Cretu et al., 2008) was an open-label, randomized, non-placebo-controlled trial; however, we did not use data from the Cretu study (Cretu et al., 2008), because these data were unavailable for the meta-analysis.

### Results of Meta-Analysis in Terms of Primary Outcomes

**Figure 1.**
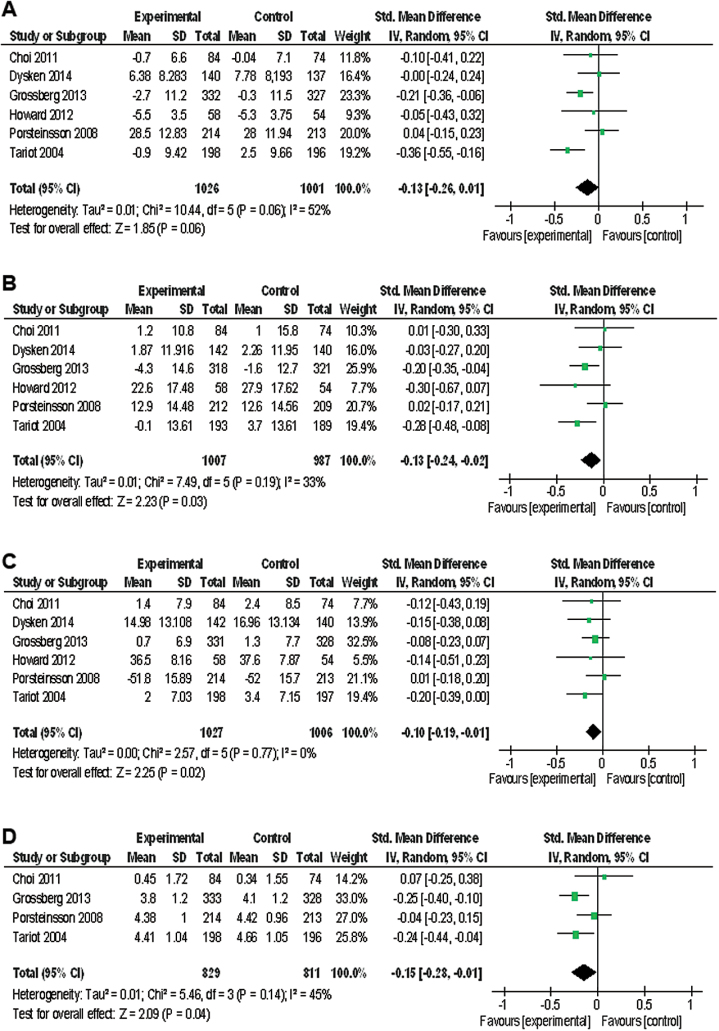
Forest plot of cognitive function, neuropsychiatric inventory (NPI), activity of daily living, and global assessment. (a) Cognitive function (prior to Alzheimer’s Disease Assessment Scale cognitive subscale [ADAS-cog], 6 studies, n=2027). (b) NPI (6 studies, n=1994). (c) Activity of daily living (6 studies, n=2033). (d) Global assessment (4 studies, n=1640). CI, confidence interval.

ChEI+MEM significantly affected NPI scores (SMD=−0.13, 95% CI=−0.24 to −0.02, Z=2.23, *P=.*03, *I* ^2^=33 %, 6 studies, n=1994) (Figure 1b). In addition, cognitive function scores (SMD=−0.13, 95% CI=−0.26 to 0.01, Z=1.85, *P=.*06, *I* ^2^=52 %, 6 studies, n=2027) (Figure 1a) exhibited favorable trends with ChEI+MEM. The data in each treatment group were simulated with no publication bias (data not shown).

### Sensitivity Analyses of Primary Outcomes

There was significant heterogeneity in cognitive function scores between studies (I^2^=52%) (Figure 1a). Therefore, we performed a sensitivity analysis to determine the confounding factors (Table 2a). When divided into a mild-to-moderate AD group and moderate-to-severe AD group in the sensitivity analysis of the prior to ADAS-cog (ADAS-cog: 3 studies, SIB: 2 studies, SMMSE: 1 study), the significant heterogeneity in cognitive function scores disappeared in both subgroups (mild-to-moderate AD, *I* ^2^=0%; moderate-to-severe AD, *I* ^2^=16%) (Table 2a). In addition, with regard to cognitive function scores, there was a more significant effect of ChEI+MEM in the moderate-to-severe AD subgroup (SMD=−0.24, *P=.*0003) than was apparent in the main meta-analysis result (SMD=−0.13, *P=.*06); however, there were no significant effects of ChEI+MEM in the mild-to-moderate AD subgroup (SMD=0.00, *P=.*97) (Table 2a). In addition, the prior to MMSE analyses (MMSE: 3 studies, SIB: 2 studies, SMMSE: 1 study,) also had similar results to that of the prior to ADAS-cog (Table 2b). Moreover, when divided by neuropsychological tests in the sensitivity analysis of the prior to ADAS-cog, the significant heterogeneity in cognitive function scores disappeared in both subgroups (SIB, *I* ^2^=21%; SMMSE, *I* ^2^=not applicable; ADAS-cog, *I* ^2^=0%) (Table 2a). Furthermore, a significant effect of ChEI+MEM on cognitive function scores was found with the SIB (SMD=−0.27, *P=.*0001) but not with the SMMSE (SMD=−0.05, *P=.*77) or ADAS-cog (SMD=0.00, *P=.*97) (Table 2a). In addition, the sensitivity analysis of the prior to MMSE also had similar results to that of the prior to ADAS-cog (Table 2b).

**Table 2.** Sensitivity Analysis of Efficacy (Cognitive Function) of Combination Therapy with Cholinesterase Inhibitors and Memantine **Table 2a**. Prior to ADAS-cog

Variable	Subgroup	N	n	I^2^	SMD	95% CI	*P* value	Test for subgroup differences
Placebo-controlled or Non-placebo-controlled	Placebo-controlled	5	1869	61	-0.1	-0.28 to 0.03	.1	I^2^ = 0%, *P* = .86
	Non-placebo-controlled	1	158	na	-0.1	-0.41 to 0.22	.55	
Cholinesterase inhibitor	Donepezil	2	506	49	-0.3	-0.53 to 0.04	.09	I^2^ = 0%, *P* = .57
	Rivastigmine	1	158	na	-0.1	-0.41 to 0.22	.55	
	Others	3	1363	58	-0.1	-0.24 to 0.10	.42	
Stages of Alzheimer’s disease	Mild to moderate	3	862	0	0	-0.13 to 0.14	.97	I^2^ = 84.9%, ***P* = .01**
	Moderate to severe	3	1165	16	-0.2	-0.38 to -0.11	**.0003**	
Neuropsychological test	ADAS-cog	3	862	0	0	-0.13 to 0.14	.97	I^2^ = 74.2%, ***P* = .02**
	SMMSE	1	112	na	-0.1	-0.43 to 0.32	.77	
	SIB	2	1053	21	-0.3	-0.41 to -0.13	**.0001**	
Sample size	Total n ≥ 200	4	1757	70	-0.1	-0.31 to 0.04	.13	I^2^ = 0%, *P* = .70
	Total n < 200	2	270	0	-0.1	-0.32 to 0.16	.52	
Memantine dose	Memantine 20 mg	5	1368	56	-0.1	-0.27 to 0.07	.25	I^2^ = 0%, *P* = .33
	Memantine 28mg extended-release	1	659	na	-0.2	-0.36 to -0.06	**.007**	
**Table 2b**. Prior to MMSE								
**Variable**	**Subgroup**	**N**	**n**	**I** ^**2**^	**SMD**	**95% CI**	***P* value**	**Test for subgroup differences**
Placebo-controlled or Non-placebo-controlled	Placebo-controlled	5	1850	48	-0.2	-0.28 to -0.02	**.03**	I^2^ = 64.3%, *P* = .09
	Non-placebo-controlled	1	158	na	0.14	-0.17 to 0.45	.38	
Cholinesterase inhibitor	Donepezil	2	506	49	-0.3	-0.53 to 0.04	.09	I^2^ = 39.3%, *P* = .19
	Rivastigmine	1	158	na	0.14	-0.17 to 0.45	.38	
	Others	3	1344	33	-0.1	-0.24 to 0.03	.13	
Stages of Alzheimer’s disease	Mild to moderate	3	843	0	0.01	-0.13 to 0.14	.91	I^2^ = 85.4%, ***P* = .009**
	Moderate to severe	3	1165	16	-0.2	-0.38 to -0.11	**.0003**	
Neuropsychological test	SIB	2	1053	21	-0.3	-0.41 to -0.13	**.0001**	I^2^ = 74.9%, ***P* = .02**
	MMSE	3	843	0	0.01	-0.13 to 0.14	.91	
	SMMSE	1	112	na	-0.1	-0.43 to 0.32	.77	
Sample size	Total n ≥ 200	4	1738	59	-0.2	-0.31 to -0.01	**.04**	I^2^ = 56.6%, *P* = .13
	Total n < 200	2	270	0	0.06	-0.18 to 0.30	.63	
Memantine dose	Memantine 20 mg	5	1349	59	-0.1	-0.25 to 0.09	.36	I^2^ = 17.7%, *P* = .27
	Memantine 28mg extended-release	1	659	na	-0.2	-0.36 to -0.06	**.007**	

ADAS-cog, Alzheimer’s Disease Assessment Scale cognitive subscale, CI, Confidence interval, MMSE, Mini-Mental State Examination, SIB, Severe Impairment Battery, SMD, standardized mean difference, SMMSE, Standardized Mini–Mental State Examination.

### Results of Meta-analysis in Terms of Secondary Outcomes

ChEI+MEM significantly affected activities of daily living scores (SMD=−0.10, CI=−0.19 to −0.01, Z=2.25, *P=.*02, *I* ^2^=0 %, 6 studies, n=2033) (Figure 1c) and global assessment scores (SMD=−0.15, CI=−0.28 to −0.01, Z=2.09, *P=.*04, *I* ^2^=45 %, 4 studies, n=1640) (Figure 1d). The data in each treatment group were simulated with no publication bias (data not shown).

**Figure 2.**
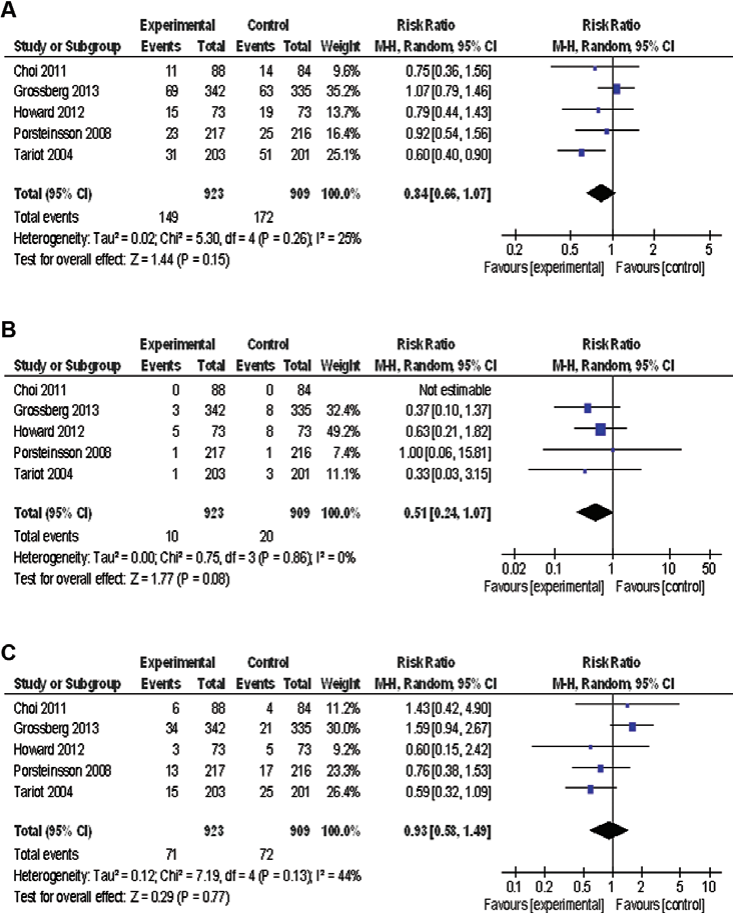
Forest plot of discontinuation rate. (a) Discontinuation due to all causes (5 studies, n=1832). (b) Discontinuation due to inefficacy (5 studies, n=1832). (c) Discontinuation due to adverse events (6 studies, n=1832). CI, confidence interval; M-H, Mantel-Haenszel.

The incidence of dropouts from all causes (Figure 2a), inefficacy (Figure 2b), or adverse events (Figure 2c) was similar between ChEI+MEM and ChEI monotherapy. No significant differences were found between groups in the incidence of any of the following: all adverse events, serious adverse events, agitation/aggression, confusion, anxiety/asthenia/depression, falls, influenza-like symptoms/upper respiratory infection, dizziness, urinary tract infection, diarrhea, and gastrointestinal symptoms.

## Discussion

This study provides an updated, comprehensive meta-analysis of ChEI+MEM for AD. The main results indicate that ChEI+MEM was superior to monotherapy with ChEI in terms of behavioral disturbances, activities of daily living, and global assessment, with a small effect size (SMD=−0.10 to −0.15). In addition, cognitive function scores exhibited favorable trends with ChEI+MEM (SMD=−0.13, *P=.*06).

**Figure 3.**
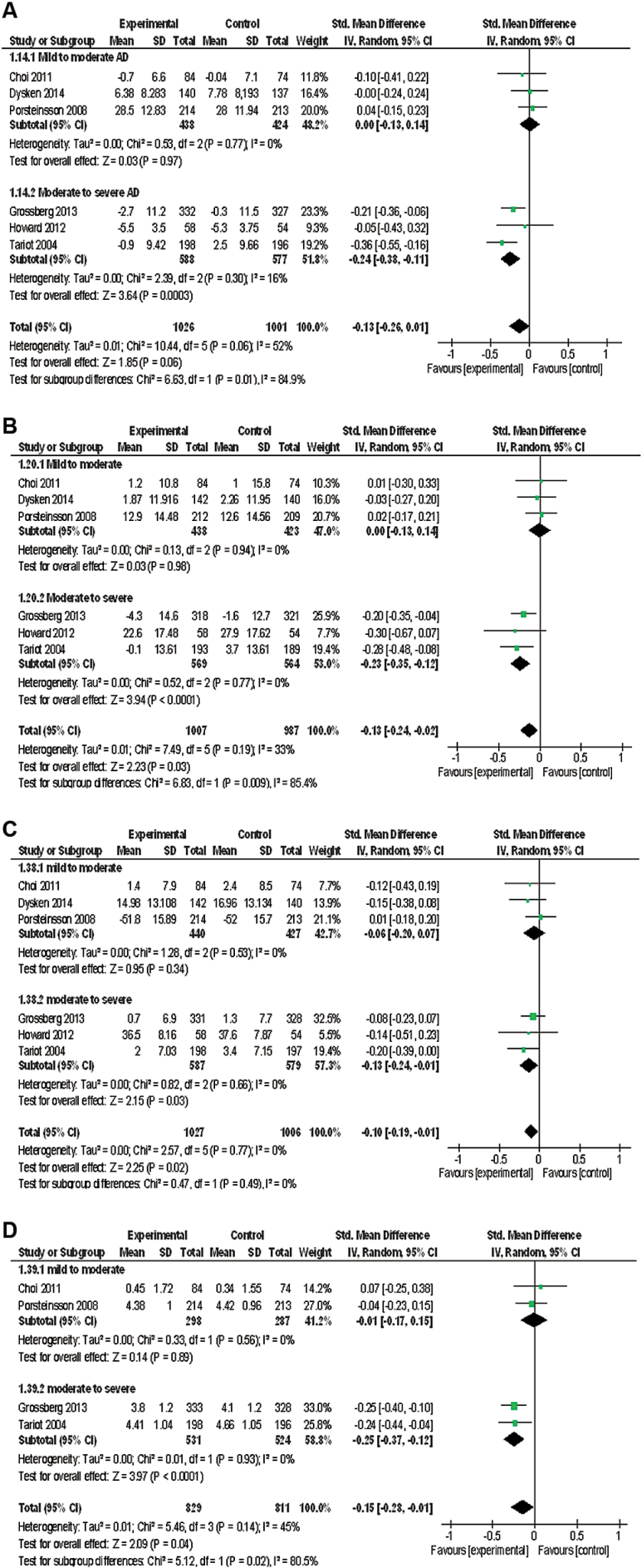
Forest plot of sensitivity and subgroup analysis (when dividing for cases where Alzheimer’s disease (AD) staging was mild to moderate or moderate to severe). (a) Cognitive function (prior to Alzheimer’s Disease Assessment Scale cognitive subscale [ADAS-cog], 6 studies, n=2027). (b) Neuropsychiatric inventory (NPI) (6 studies, n=1994). (c) Activity of daily living (6 studies, n=2033). (d) Global assessment (4 studies, n=1640). CI, confidence interval.

Sensitivity analysis revealed that heterogeneity probably resulted from AD staging and neuropsychological factors. ChEI+MEM had more significant effects on cognitive function scores in the moderate-to-severe AD subgroup, but not in the mild-to-moderate AD subgroup, than was apparent in the main meta-analysis result. Therefore, we performed subgroup analysis according to AD staging, and this trend was found for behavioral disturbance, activities of daily living, and global assessment (Figure 3).

Previous meta-analyses have reported that evidence for the efficacy of memantine for mild AD (Schneider et al., 2011) was lacking and that ChEI+MEM resulted in statistically significant but favorable changes in moderate-to-severe AD patients (Muayqil and Camicioli, 2012). Our meta-analysis was updated with a study of mild-to-moderate AD (n=479) and a study of moderate-to-severe AD (n=677). Our study supports the significant clinical benefits of ChEI+MEM, particularly for moderate-to-severe AD. In addition, it appeared that the heterogeneity in the neuropsychological tests was possibly related to AD staging, because the SIB and SMMSE were used to assess moderate-to-severe AD. On the other hand, ADAS-cog and MMSE were used to assess mild-to-moderate AD.

There were no significant differences in the rates of discontinuation from all causes and side effects between the ChEI+MEM and ChEI monotherapy groups. The study published by Dysken and colleagues (Dysken et al., 2014) was not included in the discontinuation analysis, because this study did not have a fixed duration of study (6 months to 4 years). However, there were no significant differences in the rates of discontinuation from all causes between the ChEI+MEM and ChEI monotherapy groups in their study. From the above results, ChEI+MEM does not appear to worsen the symptoms of AD and appears to be well tolerated, thus giving the impression of decreasing the severity of AD.

In a recent study, Hager et al. (2014) reported that 437 patients taking memantine and 1375 AD patients not taking this drug were randomly assigned to galantamine or placebo and were followed-up for 2 years. In posthoc analysis of this study, among patients not taking memantine, the galantamine group showed a 1.12-point decrease on MMSE and the placebo group showed a 2.15-point decrease. Among patients taking concomitant memantine, the galantamine group showed a 2.35-point decrease and the placebo group showed a 2.10-point decrease. Therefore, galantamine reduced cognitive decline by 1.03 points in the absence of memantine, but among patients taking concomitant memantine, galantamine had no effect. This discrepancy in the results may be able to explain that galantamine has a positive allosteric modulator of nicotinic receptors, increasing the open time of receptors (Williams et al., 2011). In contrast, memantine has potential to act as an open-channel blocker of nicotinic receptors (Pandya and Yakel, 2011). Memantine levels in brain tissue are markedly greater than those in the plasma and primarily represent the bound form, because cerebrospinal fluid levels are low (Ametamey et al., 2002). Memantine binding is greatest in the thalamus, followed by the striatum, cortex, and frontal white matter, with levels increasing and not yet plateauing 2 hours after administration (Ametamey et al., 2002). According to positron emission tomography study of ^18^F-memantine in healthy volunteers, there are no NMDA receptors in the white matter. Labeled 5-I-A-85380 (a nicotinic receptor ligand) binds to the human brain with the same distribution as that of as ^18^F-memantine: the highest in the thalamus, followed by the striatum and cortex, and then the white matter (Pimlott et al., 2004). From the results, because ^18^F-memantine seemed to label nicotinic receptors, memantine might be indicated to block brain nicotinic receptors during clinical use. In a smoking experiment, memantine blocks the “buzz” that smokers experience after a cigarette (Jackson et al., 2009). Accordingly, memantine-induced abolition of the cognitive benefit of galantamine would be consistent with nicotinic blockade. Therefore, when we use memantine as add-on therapy, it may be important that we consider which ChEI is used, particularly when using memantine as add-on therapy to galantamine. Nevertheless, because there are no RCTs including only patients taking galantamine, we did not conduct a sensitivity analysis of only patients taking galantamine. Further RCTs comparing the effects of the combinations of donepezil–memantine, galantamine–memantine, and rivastigmine–memantine are required.

The first limitation is that, although a funnel plot for primary and secondary outcomes did not suggest the presence of publication bias, the number of studies included in the meta-analysis was small to allow any reasonable interpretation of the funnel plots. A second limitation is that patients with dementia are known to have poor compliance with medication regimens (Boada and Arranz, 2013); therefore, the effectiveness of pharmacological interventions may be limited in this group. Finally, because several studies included in the meta-analysis did not report any available data of the symptom scales and safety outcomes in their articles, the outcome results for efficacy and safety did not include data from all the studies included in this meta-analysis. In conclusion, our results suggest that ChEI+MEM is beneficial for the treatment of moderate-to-severe AD in terms of cognition, behavioral disturbances, activities of daily living, and overall impression. Furthermore, ChEI+MEM appears to be well tolerated. Therefore, we recommended that ChEI+MEM for the treatment of patients with moderate-to-severe AD. However, the ChEI–memantine interactions were unclear. To resolve this clinical problem, further RCTs with a larger sample are required.

## Statement of Interest

No grants or other funding sources were received for this study. Dr. Matsunaga has received speaker’s honoraria from Eisai, Janssen, Novartis, Daiichi Sankyo, Ono, Eli Lilly, Takeda, and Otsuka. Dr. Kishi has received speaker’s honoraria from Abbott, Astellas, Daiichi Sankyo, Dainippon Sumitomo, Eisai, Eli Lilly, GlaxoSmithKline, Yoshitomi, Otsuka, Meiji, Shionogi, Janssen, Novartis, Tanabe-Mitsubishi, and Pfizer. Dr. Iwata has received speaker’s honoraria from Astellas, Dainippon Sumitomo, Eli Lilly, GlaxoSmithKline, Janssen, Yoshitomi, Otsuka, Meiji, Shionogi, Novartis, and Pfizer. All authors declare that they have no direct conflicts of interest relevant to this study.
